# Content analysis of tobacco imagery in popular music Videos, 2018–2021

**DOI:** 10.1016/j.pmedr.2023.102188

**Published:** 2023-03-25

**Authors:** Jessica M. Rath, Brenda Dimaya, Katie M. O'Connor, Jennifer M. Kreslake, Donna M. Vallone, Elizabeth C. Hair

**Affiliations:** a Schroeder Institute at Truth Initiative, Washington, DC, USA; b Department of Health, Behavior and Society, Johns Hopkins Bloomberg School of Public Health, Baltimore, MD, USA; c Department of Social and Behavioral Sciences, NYU College of Global Public Health, New York University, New York, NY, USA

**Keywords:** Tobacco, Vaping, Youth/Young Adults, Media, Prevention

## Abstract

Exposure to tobacco imagery in movies and television has been identified as a key factor to youth smoking initiation (Davis, 2008; Bennett et al., 2020). This study aims to investigate the prevalence of tobacco imagery in popular music videos from 2018 to 2021. The weekly top 10 songs of 2018–2021 were identified using Billboard Charts categories (Hot 100, Hot R&B/Hip-Hop, Country, Rock & Alternative, Dance/Electronic, Pop Airplay). Content analyses of the top music videos using the Thumbs up Thumbs Down methodology were conducted to identify tobacco depictions on screen. The sample consisted of 1,008 music videos across four years; 196 of which contained tobacco imagery (19.4%). Between 2018 and 2021, the proportion of videos with tobacco imagery ranged between 12.8% and 23.0% of the total year sample. Tobacco incidences almost doubled from 280 occurrences in 2018 to 522 occurrences in 2020, but decreased by over half to 290 occurrences in 2021. Tobacco incidences varied by year and genre, with Hot 100 being the top offending genre in 2018 with 40.0% of videos containing tobacco imagery while Hot R&B/Hip-Hop was the top offending genre from 2019 to 2021 (52.7%, 52.5%, and 23.9% respectively). Cigarettes were most pervasive in 2019 (70.1% of total tobacco incidences), 2020 (45.6%), and 2021 (64.1%) music videos. Pipes were the most pervasive product in 2018 music videos (39.6%). Given the reach and frequency of exposure to music videos among young people, reducing tobacco imagery in popular music videos may prevent tobacco use among young people.

## Highlights

• Exposure to tobacco imagery through films and episodic programs contribute to tobacco use and initiation among young people.
• Prevalence of tobacco imagery and in popular music videos across multiple genres have not been well researched.
• Results highlight the prevalence of tobacco imagery in music videos that are very popular with youth and young adults.
• There is a need for efforts to reduce exposure to tobacco imagery within youth-oriented entertainment.

## 1 Introduction

Tobacco use among youth and young adults continues to be a critical public health concern. In 2022, 16.5 % of high school students and 4.5 % of middle school students reported current use of at least one tobacco product (Park-Lee et al., 2022). In the last decade, e-cigarettes have replaced cigarettes as the most commonly used product. In 2022, 14.2 % of 10th graders and 20.7 % of 12th graders reported current e-cigarette use (Miech et al., 2022). Comparatively, 14.0 % of 10th graders and 16.2 % of 12th graders reported current e-cigarette use in 2015 (Monitoring the Future, 2015). This rise in e-cigarette use in youth and young adults has established a new generation of young people that are susceptible to nicotine addiction and at risk of progression to combustible tobacco use (Hair et al., 2021, Hair, et al., 2021). It is well-established that exposure to tobacco product imagery across entertainment platforms has been shown to contribute to youth smoking initiation; with multiple studies indicating a causal pathway between exposure to depictions of smoking in movies and increased odds of smoking initiation in youth and young adult populations (Cin, et al., 2013, Davis, 2008, Morgenstern, et al., 2013, Primack, et al., 2008, Primack, et al., 2012). More recent research indicates a dose–response relationship between exposure to tobacco in episodic programming and e-cigarette use initiation (Bennett et al., 2020). While e-cigarettes are more widely used by youth and young adults compared to combustible products, a study by Bennett et al. (2020) demonstrates that exposure to cigarette and other combustible tobacco product imagery is associated with higher odds of e-cigarette use initiation.

Young people spend increasing amounts of time viewing content across a variety of platforms and devices. A 2019 report by Common Sense Media found that US teens, between the ages of 13 and 18 years, were on screens nearly-seven-and-a-half hours every day for entertainment (Common Sense Media, 2019). Specifically, online video viewing on YouTube and other similar platforms has increased dramatically over the last few years. Not only are increasing numbers of adolescents reporting watching videos daily, but they are also reporting longer periods of time spent watching videos. These trends have likely been exacerbated by the COVID-19 pandemic. In 2020, a study of viewing habits among US adolescents during the COVID-19 pandemic found that the mean daily screen use time was 7.7 h per day (Nagata et al., 2021). In 2018, 85 % of US teenagers specifically reported using the video website, YouTube, with 32 % reporting that YouTube was their most frequented online platform (Anderson and Jiang, 2018). In 2021, young adults were reporting even higher viewing rates of YouTube, with 95 % of adults aged 18 to 29 years reporting use of YouTube (Anderson and Jiang, 2018; Auxier & Anderson, 2021). Between January 1st, 2021, and December 31st, 2021, the top two YouTube search words were “song” and “songs” (Hootsuite, 2022). When utilizing the word “song” on the YouTube search engine followed by a specific song name, music videos of that song appear in search results, suggesting that YouTube is commonly used as a platform for viewing music videos. Music videos on YouTube can garner over 1 billion views each (King, 2019).

Youth and young adults are exposed to tobacco product imagery in music videos. A recent paper by Albert et al. (2022) conducted a comprehensive content analysis of tobacco, alcohol, and marijuana imagery in music videos popular between 2014 and 2020. Songs were chosen from the “Hot 100” list on Billboard Charts and each song was later placed into a genre. However, less is known about tobacco imagery incidences within top genres. Previous research investigating tobacco imagery in music videos has either focused on product-specific content analyses, genre-specific videos, or music videos that were popular before 2018. A study by Escobedo et al. (2021) examined the prevalence of e-cigarette imagery in 2018 Billboard Hot 100 songs and found that 3.8 % of the study sample contained e-cigarette-specific imagery. Allem et al. (2019) investigated the tobacco marketing of a specific e-cigarette brand, KandyPens, in music videos. Results found that KandyPens products appeared in 29 popular music videos that had over 1 billion views in total on YouTube. Genre-specific music video research by Knutzen et al. (2018) found that over 40 % of popular hip-hop music videos in 2015 and 2016 contained tobacco imagery. In contrast to these previous studies, the current study provides a more in-depth analyses of tobacco imagery within each genre, increasing the total sample size.

Two recent studies explored the effects of exposure to music videos containing e-cigarette product imagery on e-cigarette use and intentions to use. Most recently, Donaldson et al. (2022) examined the impact of e-cigarette product placement in music videos on young adults’ susceptibility to use e-cigarettes. The sample of young adults watched either music videos with e-cigarette imagery or the same music videos with e-cigarette imagery digitally removed. Results found that those who watched music videos with e-cigarette imagery were significantly more likely to report intentions to try e-cigarettes in the future (Donaldson et al., 2022). An earlier study by Majmundar et al. (2021) found that young adults who self-report exposure to music videos that were previously found to contain e-cigarette-specific imagery were more likely to report lifetime e-cigarette use and past 30-day e-cigarette use compared to those with no exposure.

This study sought to examine the prevalence of all tobacco imagery (cigarettes, cigars, pipes, smokeless products) and e-cigarettes in popular music videos from 2018 to 2021 across multiple music genres. Among the music videos containing tobacco imagery, this study also sought to explore record labels to uncover those with the most frequent tobacco imagery, as well as YouTube viewership statistics to serve as a measure of tobacco imagery exposure.

## 2 Methods

### 2.1 Study sample

The study sample consisted of music videos for the top songs from 2018 to 2021. Top songs were determined by utilizing Billboard, a website and magazine that is the music industry standard record chart for the United States. Billboard Charts determines relative popularity of songs through a combination of airplay, download sales, streaming data, and YouTube views (Billboard, n.d.). Charts are released weekly. The list of songs analyzed was formulated by combining the top 10 songs each week in each calendar year under the categories and genres of: Hot 100, Hot R&B/Hip-Hop, Hot Country, Hot Rock & Alternative, Hot Dance/Electronic, and Pop Airplay. These genres were chosen for their wide-ranging nature and popularity. Accompanying music videos for these songs were located through the YouTube search engine. Only studio produced music videos were included in analyses, to prevent the coding of fan-made videos. Of the 1,211 top songs identified in 2018 (n = 272), 2019 (n = 305), 2020 (n = 315), and 2021 (n = 319), a total of 1,008 had accompanying music videos.

### 2.2 Analysis

The 1,008 music videos in the sample were reviewed by two independent coders who watched each music video in full length using the Thumbs Up Thumbs Down Methodology. The methodology was adapted from previous work investigating tobacco imagery in episodic television and movies (Cullen et al., 2011). This method records the number of tobacco occurrences by the following criteria: (1) product must be visible, (2) when two characters are smoking, this is considered two occurrences, (3) product can be lit or unlit, (4) when one character is holding multiple products, each product is considered a separate occurrence, (5) each time a product is moved off the screen or out of focus and then back on screen or into focus, this is two separate occurrences, and (7) a carton of cigarettes or a pack of any type of product is counted as one occurrence. Products were coded as: cigarettes, cigars, pipe tobacco (including hookah and bong), smokeless products, or e-cigarettes. A category named “indistinguishable blunt/cigar” was added to represent products that couldn’t be determined due to the fast-moving nature of music videos. This category largely consisted of products that could have either been marijuana in a tobacco leaf wrapper or tobacco-only little cigars and cigarillos. Researchers also coded for tobacco product placement including visible brand names and how the brands were depicted. Music videos with discrepancies in coding were resolved by a third coder. Analyses of tobacco occurrences by genre was also completed. Some songs are not exclusive to one genre, and 58 songs fell into more than one genre. To estimate reach and serve as a proxy for exposure, YouTube viewership statistics were obtained for the songs where tobacco use was present. YouTube viewership statistics were all obtained on the same date to improve comparability between videos. Members of the study team looked up each song on Discogs, a database that identifies the record label for individual songs, along with other contextual information such as the performing artist, release date, and recording studio (Discogs, n.d.).

## 3 Results

### 3.1 Coding results

Table 1 presents the findings by tobacco product type and genre in top music videos of 2018–2021. The sample consisted of 1,008 music videos across four years and in total, 196 videos contained tobacco imagery (19.4 %). In 2018, of the 236 music videos analyzed, 49 contained tobacco imagery (20.8 %). Across the 49 videos, there were 280 tobacco occurrences. Among the 272 top music videos coded in 2019, 57 music videos contained tobacco imagery (21.0 %). Across the 57 videos, there were 462 tobacco occurrences. In 2020, 59 out of 257 music videos contained tobacco imagery (23.0 %) and across the 59 videos, there were 522 tobacco occurrences. In 2021, 31 of the 243 music videos contained tobacco imagery (12.8 %). Overall, 290 incidences of tobacco imagery appeared across the 31 music videos.

**Table 1:** Total Tobacco Incidences in Music Videos Genre by Product, 2018–2021.

Genre	Year	Songs with music videos	Music videos with tobacco n(%)**	Total tobacco incidences	Cigarettes	Indistinguishable Blunt/Cigar	Cigars	Pipes	E-cigarettes	Smokeless
Country	2018	52	8 (15.4)	22	12	5	2	2	1	0
	2019	56	3 (5.4)	12	12	0	0	0	0	0
	2020	48	3 (6.3)	13	3	0	10	0	0	0
	2021	59	4 (6.8)	9	6	0	0	3	0	0

Dance/Electronic	2018	39	2 (5.1)	5	5	0	0	0	0	0
	2019	42	4 (9.5)	111	106	1	0	0	0	4
	2020	43	3 (7.0)	52	46	6	0	0	0	0
	2021	42	3 (7.1)	60	55	5	0	0	0	0

Hot R&B /Hip-Hop	2018	59	23 (39.0)	161	34	17	34	62	14	0
	2019	55	29 (52.7)	261	144	55	60	0	2	0
	2020	61	32 (52.5)	326	110	184	21	9	2	0
	2021	67	16 (23.9)	168	134	25	8	0	1	0

Hot Rock & Alternative	2018	53	10(18.9)	22	13	5	1	3	0	0
	2019	81	15 (18.5)	58	44	6	1	0	7	0
	2020	78	18 (23.1)	118	74	36	3	1	4	0
	2021	45	5 (11.1)	47	44	3	0	0	0	0

Pop Airplay	2018	48	11 (22.9)	97	31	2	20	31	13	0
	2019	50	11 (22.0)	60	53	5	1	0	1	0
	2020	49	8 (16.3)	83	45	15	21	0	2	0
	2021	39	2 (5.1)	3	0	3	0	0	0	0

Hot 100	2018	65	26 (40.0)	218	45	16	37	106	14	0
	2019	58	20 (34.5)	210	138	12	59	0	1	0
	2020	59	17 (28.8)	117	38	72	6	0	1	0
	2021	52	9 (17.3)	143	125	12	6	0	0	0

Total	2018	236	49 (20.8)	280	75	28	51	111	15	0
	2019	272	57 (21.0)	462	324	63	61	0	10	4
	2020	257	59 (23.0)	522	238	211	57	10	6	0
	2021	243	31 (12.8)	290	186	34	11	58	1	0

*Songs may fall into multiple genres; tobacco incidences do not add up to column totals.

**Percentage of total songs with music videos within each genre.

Tobacco occurrences in music videos almost doubled from 280 tobacco occurrences in 2018 to 522 tobacco occurrences in 2020. Tobacco occurrences decreased by almost half from 522 total tobacco incidences in 2020 to 290 tobacco incidences in 2021. However, tobacco incidences are higher in 2021 compared to 2018. From 2019 to 2021, cigarettes were the most pervasive tobacco product, accounting for 70.1 % of total tobacco occurrences in 2019, 45.6 % in 2020, and 64.1 % in 2021. In 2018, pipes, which mostly consisted of hookah, were the most pervasive tobacco products, accounting for 39.6 % of tobacco occurrences. In 2020, the “indistinguishable blunt/cigar” category was higher compared to other years, comprising 40.4 % of total tobacco occurrences.

E-cigarette and smokeless product depictions were low across all years, comprising less than 5 % of tobacco occurrences each year. 2018 had the most e-cigarette depictions, compromising 5.4 % of total tobacco occurrences. Smokeless tobacco products were only identified in 2019.

Tobacco occurrences by genre varied by year. In 2018, the Hot 100 was the top offending genre with 40 % of music videos containing tobacco imagery. The genre, Hot R&B/Hip-Hop became the top offending genre from 2019 to 2021. Between 2019 and 2021, the proportion of videos with tobacco imagery in the Hot R&B/Hip Hop genre ranged between 23.9 % and 52.7 % of total music videos.

### 3.2 Viewership and record labels

YouTube viewership statistics were obtained to serve as a measure for exposure to tobacco imagery. As of June 6th, 2022, among all music videos where tobacco imagery was present, there were a total of over 38 billion views on YouTube. The most popular music videos containing tobacco imagery by viewership had over 1 billion views each (“Life is Good” by Future (Future, 2020), “Sunflower” by Post Malone & Swae Lee (Post Malone, 2019), “I Like It” by Cardi B (Cardi et al., 2018), “Mia” by Bad Bunny (Bad Bunny, 2019), “Shallow” by Lady Gaga (Lady Gaga, 2018). Viewership ranged from 1,009,192,557 to 2,033,133,463 with an average viewership of 1,349,033,111. The average length of a music video was 3 min and 56 s. The longest video was 8 min and 5 s while the shortest was 2 min and 26 s. Three songs “Smokin Out the Window” by Bruno Mars & Anderson. Paak (Bruno Mars, 2021), “Think About You” by Kygo & Valerie Broussard (Kygo, 2019), and “El Incomprendido” by Farruko, Victor Cardenas, & DJ Adoni (Farruko, 2021), had over 50 incidences of tobacco imagery each in music videos that were less than five minutes in length (3:21, 4:34, and 4:29 min respectively).

Record labels were also identified for each music video that contained tobacco to identify which label produced the most music videos with tobacco imagery. There was a total of 88 record labels identified. The record label company Atlantic Records was the top offender, with 14 music videos containing 214 tobacco occurrences across 2018 to 2021 (see Table 2). Republic Records, Interscope Records, Epic Records, and Sony Music Entertainment followed as top offenders. The top 5 offenders were responsible for over 100 tobacco occurrences.

**Table 2:** Top 10 Offending Record Labels by Total Music Videos with Tobacco Imagery, 2018–2021.

Total Music Videos with tobacco imagery *	Record Label	Total Tobacco Incidences	Average of tobacco incidences per video
26	Republic Records	181	7.0
25	Interscope Records	180	7.2
18	Epic Records	138	7.7
14	Atlantic Records	214	15.3
10	Columbia Records	62	6.2
8	Grade A Productions	85	10.6
5	Sony Music Entertainment	138	27.6
2	Aftermath Records	112	56
2	Ultra Records	58	29
1	Sony Music Entertainment US Latin	55	55

*Some songs have multiple record labels.

### 3.3 Brands

We identified two tobacco product brands, KandyPens and Maverick cigars, in this study. KandyPens were identified in the music video, “No Brainer” by DJ Khaled ft. Justin Bieber, Chance the Rapper, and Quavo (DJ Khaled, 2018), in which the products were clearly visible in multiple scenes and were smoked by the cast, including the artist himself. A poster of Maverick was placed in a convenience store in the music video “Straightenin” by Migos (Migos, 2021).

## 4 Discussion

Tobacco imagery is prevalent in popular music videos. We found that nearly-one-fourth of popular music videos from 2018 to 2020 across multiple genres contained tobacco imagery, with the proportion of music videos containing tobacco increasing over time. Although the proportion of music videos with tobacco imagery dropped by approximately half in 2021, tobacco imagery is still pervasive. Imagery is so pervasive in some videos that it results in exposure to tobacco imagery approximately every-five seconds in such videos. It may be that the decline in 2021 is not representing systemic change but rather other factors (ie. production issues resulting from COVID-19) could be contributing to the observed findings. Further monitoring is warranted.

While tobacco incidences occurred in all genres, the hip-hop genre was the biggest offender, accounting for over half of tobacco incidences from 2019 to 2021. This matches previous studies that found that tobacco imagery was present in up to half of hip-hop songs popular between 2013 and 2017 (Knutzen et al., 2018). The current study suggests that little has changed, with a similar proportion of videos found in this genre containing tobacco imagery between 2018 and 2021. This is especially concerning given the reach of hip-hop music among racial and ethnic minorities who are already at greater risk of combustible tobacco use (Nielsen. , 2018, Wang, et al., 2019).

Despite a rise in e-cigarette use in the United States, e-cigarette imagery was not predominant in the current study, in contrast to findings from previous studies which detailed e-cigarette brand placements in music videos (Allem et al., 2019, Knutzen, et al., 2018; Donaldson et al., 2022; Majmundar et al., 2021). While some research has noted a prominent instance of e-cigarette brand placement in music videos as early as 2015 (National Center for Chronic Disease Prevention and Health Promotion (US) Office on Smoking and Health, 2016), the current study only identified e-cigarette brand placement for KandyPens in the 2018 music video, “No Brainer” by DJ Khaled ft. Justin Bieber, Chance the Rapper, and Quavo (DJ Khaled, 2018).

The decrease in e-cigarette imagery and visible tobacco product brands in videos may be linked to the 2018 KandyPens lawsuit filed by Los Angeles. The lawsuit alleged that KandyPens was marketing their tobacco products to youth through social media platforms and product placements in music videos, including the video “No Brainer” by DJ Khaled ft. Justin Bieber, Chance the Rapper, and Quavo (DJ Khaled, 2018; Berg, 2021). In 2021, KandyPens was banned from marketing its products to youth and faced financial penalties. The lawsuit may have deterred tobacco companies and producers from placing visible brand products in music videos to avoid similar penalties. Future research that monitors combustible tobacco and e-cigarette in media such as music videos should include a coding category for brand placements featuring recognizable product names, packaging, or logos to identify marketing strategies by specific e-cigarette companies that can reach youth and young adult audiences.

Youth and young adults are particularly vulnerable to exposures to tobacco and e-cigarette use modeled by popular media figures or depicted in an appealing or glamorous way. Exposure to both combustible tobacco and non-combustible imagery on screens is associated with an increased likelihood of vaping initiation (Bennett et al., 2020; National Center for Chronic Disease Prevention and Health Promotion Office on Smoking and Health, 2016). While the current study did not find extensive e-cigarette imagery in music videos, results found pervasive amounts of combustible tobacco products. Therefore, the tobacco imagery found in this study poses a substantial risk to the tobacco and e-cigarette use behavior of youth and young adults.

Young audiences drive much of the popularity of artists and songs, and thus are key consumers of music videos. Although YouTube viewership numbers do not indicate viewership statistics by age group, previous research has found that the majority of youth and young adults use YouTube (Anderson and Jiang, 2018). Additionally, according to Pew Research, the YouTube recommendation algorithm that generates suggested videos to users progressively pushes users to watch longer videos (van Kessel, 2021) and videos of similar content which could exponentially increase youth tobacco exposure.

The pervasiveness of onscreen tobacco imagery points to an overall problem with the normalization and glamorization of tobacco in entertainment media and pop culture. Several measures can be taken to prevent youth exposure to tobacco imagery in music videos. States can reduce tobacco content in music videos by changing film subsidy policies and other incentives to exclude productions with tobacco content. Presently, more than 30 states offer subsidies, tax breaks, and other incentives to encourage production companies to film in-state (Nguyen, 2021). In some states, music videos are eligible for state incentives (Hawaii Film Office, 2011; Georgia Film, 2021). State subsidies and incentives may be leveraged to exclude productions with tobacco content from access to incentives.

Record labels are responsible for the content they produce and can play a key role in reducing tobacco imagery in music videos. Thus, one approach to protecting youth and young adults from tobacco imagery on screens is for record labels to make the creative decision to remove tobacco content from their videos. Another approach is to involve record labels in assigning ratings to music videos to provide guidance to guardians on suitable content to minors. Of all the music videos coded in this study, only one video was rated with two versions: one video rated PG and one video rated R. However, the PG-rated version still contained tobacco imagery. Finally, streaming sites such as YouTube should age restrict music videos with tobacco to those over the age of 21. Currently, age-restricted videos on YouTube can only be accessed by users who have verified that they are over 21 years old (YouTube, 2021).

## 5 Limitations

This study is not without limitations. There are songs, which may be popular with youth and young adults, that have been excluded because they did not make it onto Billboard Charts. Meeting the criteria for Billboard may be difficult for unsigned and new artists. Therefore, this study does not capture the amount of tobacco imagery in all popular music videos and may underrepresent the full scope of exposure to tobacco imagery. However, this study coded the top 10 songs on Billboard charts each week to be comprehensive of the top videos. In addition to coding music videos, this study also coded for references to tobacco products in lyrics. We found few references to tobacco products in lyrics and results were not included to maintain the focus of this paper on visual imagery.

While this study reported on YouTube viewership, it was beyond the scope of the research to report on viewership of music videos on other potential viewing platforms. This viewership also did not provide age or other demographic information which poses challenges in estimating tobacco imagery exposure in youth and young adult populations. Although we coded for record labels, we are unable to provide an accurate measure of tobacco occurrences for each label because not all of their songs made it to the Billboard top charts.

## 6 Conclusion

Tobacco on screens is contributing to youth use and initiation (Davis, 2008, Bennett et al., 2020). The expansion of the media landscape and increasing screen time among young people means that there are more opportunities for exposure than ever before. We know that glamorizing tobacco use on screens leads to the normalization of tobacco products. There is no evidence that showing tobacco on screens is benefiting the artist or record label in any way. Instead, the artists and record labels are providing free advertising for Big Tobacco by becoming their unpaid spokespeople. The study by Bennett et al. (2020) also found that exposure to tobacco imagery through episodic programming can triple a young person’s odds of starting to vape. Additionally, people with more exposure to tobacco in movies are twice as likely to begin smoking (National Center for Chronic Disease Prevention and Health Promotion (US) Office on Smoking and Health, 2016).

The music industry needs to be held accountable for exposing youth and young adults to tobacco imagery from their content. The results of this study highlight the prevalence of tobacco imagery in music videos that are very popular with youth and young adults. Reducing exposure to tobacco imagery within youth-oriented entertainment is critical because it may start to de-normalize and prevent tobacco use among young people.

## Funding

Evaluation support: Tobacco Use in Popular Media Targeted to Youth (CDC Req# 75D301-20-Q-71818) and Truth Initiative Internal Funding.

## CRediT authorship contribution statement

**Jessica M. Rath:** Supervision, Methodology, Writing – original draft, Writing – review & editing. **Brenda Dimaya:** Methodology, Formal analysis, Writing – original draft, Investigation. **Katie M. O'Connor:** Methodology, Formal analysis, Writing – original draft, Investigation. **Jennifer M. Kreslake:** Methodology, Writing – review & editing. **Donna M. Vallone:** Writing – review & editing. **Elizabeth C. Hair:** Supervision, Methodology, Writing – review & editing.

## Declaration of Competing Interest

The authors declare that they have no known competing financial interests or personal relationships that could have appeared to influence the work reported in this paper.

## Data Availability

Data will be made available on request.

## References

[b0005] Albert S.L., Rogers E., Hall Z., Zuardo G., Bragg M.A. (2022). Comparing the Prevalence of Alcohol, Combustible and Electronic Cigarettes, Hookah, and Marijuana, in Music Videos across 6 Genres of Popular Music from 2014–2020. Subst. Use Misuse.

[b0010] Allem J.-P., Escobedo P., Cruz T.B., Unger J.B. (2019). Vape pen product placement in popular music videos. Addict. Behav..

[b0015] Anderson M., Jiang J. (31 May 2018,).

[b0025] Auxier, Brooke and Anderson, Monica. Social Media Use in 2021. 22 Nov. 2021, https://www.pewresearch.org/internet/2021/04/07/social-media-use-in-2021/. Accessed November 21, 2021.

[b0030] BAD BUNNY x DRAKE - MÍA (Official Video). Directed by Bad Bunny, 2018. YouTube, https://www.youtube.com/watch?v=OSUxrSe5GbI. Accessed November 7, 2022.

[b0035] Bennett M., Hair E.C., Liu M., Pitzer L., Rath J.M., Vallone D.M. (2020). Exposure to Tobacco Content in Episodic Programs and Tobacco and E-Cigarette Initiation. Prev. Med..

[b0040] Berg L. (19 Apr. 2021,). LA City Atty Gets $1.2M Penalty, Injunction Against Vape Co. Law.

[b0045] Billboard. Billboard Charts Legend. https://www.billboard.com/billboard-charts-legend/. Accessed December 7, 2021.

[bib216] Bruno Mars - Smokin Out The Window [Official Music Video]. Directed by Bruno Mars, 2021. YouTube, https://www.youtube.com/watch?v=GG7fLOmlhYg. Accessed November 21, 2022.

[b0050] Cardi B, Bad Bunny & J Balvin - I Like It [Official Music Video]. Directed by Cardi B, 2018. YouTube, https://www.youtube.com/watch?v=xTlNMmZKwpA. Accessed November 7, 2022.

[b0055] National Center for Chronic Disease Prevention and Health Promotion (US) Office on Smoking and Health. E-Cigarette Use Among Youth and Young Adults: A Report of the Surgeon General. Centers for Disease Control and Prevention (US), 2016. PubMed, http://www.ncbi.nlm.nih.gov/books/NBK538680/. 30869850

[b0060] Dal Cin, Sonya, et al. “Exposure to Smoking in Movies and Smoking Initiation Among Black Youth.” American Journal of Preventive Medicine, vol. 44, no. 4, Apr. 2013, pp. 345–50, 10.1016/j.amepre.2012.12.008. 10.1016/j.amepre.2012.12.008 PMC3674583 23498099

[b0065] Common Sense Media (2019). The Common Sense Census: Media Use by Tweens and Teens. Common Sense. Media.

[b0070] Cullen J., Sokol N.A., Slawek D., Allen J.A., Vallone D., Healton C. (2011). Depictions of Tobacco Use in 2007 Broadcast Television Programming Popular Among US Youth. Arch. Pediatr. Adolesc. Med..

[b0075] Davis, Ronald M. The Role of the Media in Promoting and Reducing Tobacco Use. US Department of Health and Human Services, National Institutes of Health, National Cancer Institute, 2008. 10.1037/e481902008-001.

[b0080] Discogs. https://www.discogs.com/. Accessed December 7, 2021.

[bib217] DJ Khaled – No Brainer (Office Video) ft. Justin Bieber, Chance the Rapper, Quavo. Direct by DJ Khaled, 2018. YouTube, https://www.youtube.com/watch?v=kxloC1MKTpg. Accessed November 21, 2022.

[b0085] Donaldson, S.I., Dormanesh, A., Escobedo, P., Majmundar, A., Kirkpatrick, M., Allem, J., 2022. “The Impact of E-Cigarette Product Place in Music Videos on Susceptibility to Use e-Cigarettes among Young Adults: An Experimental Investigation.” Addictive Behaviors, vol. 130, no. 107307, 107307. 10.1016/j.addbeh.2022.107307. 35305325

[b0090] Escobedo, Patricia, et al. “Electronic Cigarette Product Placement and Imagery in Popular Music Videos.” Nicotine & Tobacco Research: Official Journal of the Society for Research on Nicotine and Tobacco, vol. 23, no. 8, 8, Aug. 2021, pp. 1367–72. PubMed, 10.1093/ntr/ntaa273. 10.1093/ntr/ntaa273 33367917

[b0095] Farruko, Victor Cardenas, Dj Adoni - El Incomprendido (Official Video). Directed by Farruko, https://www.youtube.com/watch?v=Zpi7CTDvi1A. Accessed November 21, 2022.

[b0100] Georgia Film. Film Tax Incentives 2021. Georgia Department of Revenue, 2021, https://www.georgia.org/sites/default/files/2021-03/georgia_film_tax_incentive_brochure_2021_1.pdf.

[b0105] Future - Life Is Good (Official Music Video) Ft. Drake. Directed by Future, 2020. YouTube, https://www.youtube.com/watch?v=l0U7SxXHkPY. Accessed November 21, 2022.

[b0115] Hair E.C., Kreslake J.M., Mowery P., Pitzer L., Schillo B., Vallone D.M. (2021). A Longitudinal Analysis of E-Cigarette Use and Cigar, Little Cigar or Cigarillo Initiation among Youth and Youth Adults: 2017–2019. Drug Alcohol Depend..

[b0120] Hair, Elizabeth C., et al. “Association between E-Cigarette Use and Future Combustible Cigarette Use: Evidence from a Prospective Cohort of Youth and Young Adults, 2017-2019.” Addictive Behaviors, vol. 112, Jan. 2021, p. 106593. PubMed, 10.1016/j.addbeh.2020.106593. 10.1016/j.addbeh.2020.106593 32927247

[b0125] Harmful or Dangerous Content Policies - YouTube Help. https://support.google.com/youtube/answer/2801964#age_restricted&zippy=%2Cage-restricted-content. Accessed November 21, 2021.

[b0020] (Aug. 2011). Hawaii Film Office.

[b0130] Hootsuite. (Jan. 2022). Digital 2022 Global Overview Report: The Essential Guide to the World’s Connected Behaviours. HootSuite.

[b0135] King, Ashley. “Music Makes Up 5% of All Content on YouTube—And 20% of Total Views.” Digital Music News, 10 June 2019, https://www.digitalmusicnews.com/2019/06/10/youtube-music-study/#:∼:text=The%20average%20length%20of%20a,and%2016%2C397%20views%20per%20video. Accessed November 21, 2022.

[b0140] Knutzen, Kristin E., et al. “Combustible and Electronic Tobacco and Marijuana Products in Hip-Hop Music Videos, 2013-2017.” JAMA Internal Medicine, vol. 178, no. 12, 12, Dec. 2018, pp. 1608–15. Silverchair, 10.1001/jamainternmed.2018.4488. 10.1001/jamainternmed.2018.4488 PMC6583628 30326017

[b0145] Kygo, Valerie Broussard - Think About You (Official Video). Directed by KygoMusic, 2019. YouTube, https://www.youtube.com/watch?v=Fqfja0vj76s. Accessed November 21, 2022.

[b0110] Lady Gaga - Shallow (from A Star Is Born) (Official Music Video). Directed by Lady Gaga, 2018. YouTube, https://www.youtube.com/watch?v=bo_efYhYU2A. Accessed November 21, 2022.

[b0150] Majmundar, Anuja, et al. “Exposure to E-Cigarette Product Placement in Music Videos Is Associated With Vaping Among Young Adults.” Health Education & Behavior, Apr. 2021, p. 10901981211003868. SAGE Journals, 10.1177/10901981211003867. 10.1177/10901981211003867 PMC8492787 33821689

[b0155] Miech, Richard A., et al. “Monitoring the Future National Survey Results on Drug Use, 1975-2021: Volume 1, Secondary School Students.” Institute for Social Research (2022).

[b0160] Migos - Straightenin (Official Video). Directed by Migos, 2021. https://www.youtube.com/watch?v=E553AnMAvlU. Accessed November 21, 2022.

[b0165] Monitoring the Future Survey (Dec. 2015). Overview of Findings.

[b0170] Morgenstern, Matthis, et al. “Smoking in Movies and Adolescent Smoking Initiation: Longitudinal Study in Six European Countries.” American Journal of Preventive Medicine, vol. 44, no. 4, Apr. 2013, pp. 339–44, 10.1016/j.amepre.2012.11.037. 10.1016/j.amepre.2012.11.037 PMC3616269 23498098

[b0175] Nagata, Jason M., et al. “Screen Time Use Among US Adolescents During the COVID-19 Pandemic: Findings From the Adolescent Brain Cognitive Development (ABCD) Study.” JAMA Pediatrics, Nov. 2021. Silverchair, 10.1001/jamapediatrics.2021.4334. 10.1001/jamapediatrics.2021.4334 PMC8561427 34724543

[b0180] Nguyen R. (19 July 2021.). Film, TV Studios Are Offered Incentives From States After Pandemic Shutdowns. Wall Street J..

[b0185] Nielsen. (2018).

[b0190] Park-Lee E. (2022). Tobacco Product Use Among Middle and High School Students United States, 2022. MMWR Morb. Mortal. Wkly Rep..

[b0195] Post Malone D, Swae Lee O. Post Malone & Swae Lee - Sunflower. Directed by Post Malone, 2019. YouTube, https://www.youtube.com/watch?v=3E78T8h5EhA. Accessed November 7, 2022.

[b0200] Primack, Brian A., et al. “Adolescent Smoking and Volume of Exposure to Various Forms of Media.” Public Health, vol. 122, no. 4, Apr. 2008, pp. 379–89, 10.1016/j.puhe.2007.07.022. 10.1016/j.puhe.2007.07.022 PMC3001229 18206196

[b0205] Primack, Brian A., et al. “Association of Established Smoking Among Adolescents With Timing of Exposure to Smoking Depicted in Movies.” Journal of the National Cancer Institute, vol. 104, no. 7, Apr. 2012, 10.1093/jnci/djs138. 10.1093/jnci/djs138 PMC3317882 22423010

[b0210] van Kessel P. (22 Nov. 2021).

[b0215] Wang, TW, et al. “Tobacco Product Use and Associated Factors Among Middle and High School Students — United States, 2019.” MMWR. Morbidity and Mortality Weekly Report, vol. 68, 2019, pp. 1–22, 10.15585/mmwr.ss6812a1.

